# Repetitive negative thinking: transdiagnostic correlate and risk factor for mental disorders? A proof-of-concept study in German soldiers before and after deployment to Afghanistan

**DOI:** 10.1186/s40359-021-00696-2

**Published:** 2021-12-19

**Authors:** Katrin V. Hummel, Sebastian Trautmann, John Venz, Sarah Thomas, Judith Schäfer

**Affiliations:** 1 Department of Psychology, Institute of Clinical Psychology and Psychotherapy, Technische Universität Dresden, Chemnitzer Str. 46, 01187 Dresden, Germany; 2 Medical School Hamburg, Department of Psychology, Hamburg, Germany

**Keywords:** Repetitive negative thinking, Transdiagnostic, Correlate, Risk factor, Incident mental disorders, Stress

## Abstract

**Background and objectives:**

Disorder-specific forms of Repetitive Negative Thinking (RNT) are associated with multiple diagnostic categories, indicating a transdiagnostic nature. Few studies examined content-independent RNT processes across groups of diagnosed mental disorders. Moreover, theory describes RNT processes as critically involved in the etiology of mental disorders, empirical evidence however is scarce. We first tested the transdiagnostic nature by examining levels of RNT across groups of internalizing and externalizing mental disorders compared to healthy individuals and explored RNT levels in a comorbid disorder-group. Second, we examined whether RNT predicts incident psychopathology.

**Methods:**

In a sample of German soldiers (*n* = 425) scheduled for deployment in Afghanistan, we compared RNT levels between diagnosed groups with alcohol use disorders, anxiety disorders and healthy individuals cross-sectionally. Exploratory analyses were conducted comparing a comorbid disorder-group to healthy individuals and to both single-disorder-groups. Longitudinally, we examined the predictive value of pre-deployment RNT levels for incident psychopathology after deployment (*n* = 167). RNT was measured using the Perseverative Thinking Questionnaire (PTQ), DSM-IV diagnoses were assessed using the standardized Composite International Diagnostic Interview (CIDI).

**Results:**

Cross-sectional comparisons revealed that soldiers with alcohol use disorders and anxiety disorders showed significantly higher degrees of RNT compared to healthy soldiers. RNT levels in those with comorbid disorders were significantly higher compared to healthy soldiers but also compared to both single-disorder-groups. Longitudinal analyses revealed that higher levels of RNT prior to deployment were associated with a higher risk to have any incidental mental disorder after deployment. This however is only attributable to individuals with a PTQ score above a cut-off of 15.

**Conclusions:**

Findings provide evidence for RNT as a transdiagnostic correlate and a vulnerability factor for the development of mental disorders.

## Introduction

Lifetime prevalence estimates for any mental disorder range from 18 to 36% representing a global health issue with enormous societal and economic costs [1, 2]. Accordingly, there is a strong need for the identification of risk factors that may contribute to the development and maintenance of mental disorders. In general, most etiological models assume that an interplay of some kind of vulnerability and stress leads to psychopathology (diathesis-stress models; e.g., [3]). A plethora of theory and related empirical evidence suggests that dysfunctional cognitive processing may be such a vulnerability factor [4, 5].

Specifically, Repetitive Negative Thinking (RNT) described as “excessive and repetitive thinking about current concerns, problems, past experiences or worries about the future” ([6], p.192) is a promising candidate process. RNT comprises several disorder-specific expressions, e.g. rumination in depression, worry or post-event processing in anxiety or traumatic rumination in stress-related disorders.

Various cognitive-behavioral theories propose an association between disorder-specific forms of RNT and different mental disorders [7–11]. In line with these theoretical considerations, studies found that disorder-specific expressions of RNT were associated with symptomatologies, such as depression and anxiety [12–16], posttraumatic stress symptoms [17], bulimic [18] and substance use disorder symptoms [18–20]. Further, it was shown, that different forms of experimentally induced RNT, that is, worry and rumination, had similar effects on mood states [21]. And finally, reduction in RNT was shown to be predictive for symptom reduction during cognitive-behavioral therapy for anxiety as well as for depressive symptoms [22].

Due to the reported associations across multiple psychopathologies, several researchers proposed RNT to be a *transdiagnostic* cognitive processing style [6, 15, 23, 24]. According to Mansell et al. [25], a psychological process can be considered transdiagnostic if it has been assessed in clinical and nonclinical samples and is present in at least four disorders. This holds true for the most commonly studied RNT expressions, namely rumination and worry. However, at the same time it is difficult to draw conclusions about the transdiagnostic nature of RNT because in many studies disorder- and content-specific measures of RNT are applied. Consequently, some researchers have argued that focusing on the common formal processes of RNT instead of focusing on its disorder-specific content is a promising pathway in research on transdiagnostic RNT [6]. This view is empirically supported by factor analytic studies pointing towards great overlap of disorder-specific RNT variants [26, 27] and even a resulting one factor solution when disorder-specific content in items is removed [28]. Studies using structure equation modelling showed that a common RNT dimension was associated with multiple symptom outcomes [29, 30]. Further support for a common dimension of RNT comes from studies showing that the shared variance among different forms of RNT accounted for symptom severity and comorbidity of anxiety and depression more than disorder-specific RNT expressions [23, 30]. Yet, studies on the transdiagnostic nature of RNT have been mostly conducted using dimensionally assessed subclinical outcomes, which limits definite conclusions about the clinical relevance of these studies, especially since average symptom levels are often relatively low. Only few studies examined the association of content-independent RNT across diagnosed mental disorders [30–34]. To extend and replicate these findings it might be important to examine the associations between RNT and full-blown diagnoses of mental disorders and their comorbidities. Further, many studies on the transdiagnostic nature of RNT focused on anxiety and depression symptoms, both part of the internalizing disorder spectrum sharing key components in development and maintenance [35]. Research investigating the role of RNT in externalizing disorders such as substance use disorders found conflicting findings so far [20]. To our best knowledge, there are no studies comparing RNT in internalizing and externalizing disorders and thus, test another component of its transdiagnostic nature.

Accordingly, the *first aim* of the present study was to replicate and extend knowledge on the transdiagnostic nature of RNT by comparing RNT between mental disorders from distant diagnostic spectra, namely internalizing and externalizing disorders. Thus, we cross-sectionally compared RNT levels between groups with a diagnosis of anxiety disorders (i.e., representative of internalizing disorders), alcohol use disorders (i.e., representative of externalizing disorders) and healthy individuals in a high-risk sample of German soldiers. The two disorders were selected as representatives of internalizing and externalizing disorders because previous research showed that these are diagnostic categories with high prevalence among deployed German soldiers [36]. We assumed that RNT levels would be higher in both diagnostic groups compared to individuals with no lifetime disorder. Additionally, testing RNT levels in a group with comorbid disorders might be a valuable contribution to research on the transdiagnostic nature of RNT. Indeed, previous research has demonstrated positive associations between RNT levels and comorbidity (e.g., [12, 15, 30, 32]). However, again, this research relates mainly to internalizing disorders, i.e. depressive and anxiety disorders. Thus, on an exploratory basis, we tested whether RNT levels in a comorbid group including various mental disorders might be higher relative to healthy individuals and single diagnoses of anxiety and alcohol use disorders.

Next, while prospective-longitudinal studies provide strong support for RNT to be involved in the *maintenance* and *aggravation* of multiple symptomatologies [19, 37], there are some open questions in research investigating RNT processes in the *development* of psychopathology. This has several reasons: Studies demonstrating predictive value of RNT for new onset of diagnosed mental disorders mostly used disorder-specific RNT measures [18, 38]. Consequently, findings cannot necessarily be generalized to content-independent RNT. Studies that applied a content-independent RNT measure assessing trait-like RNT, however, revealed mixed findings. Raes et al. [39] found supportive evidence for its predictive role for depressive mood in a longitudinal study with students. In contrast, in another longitudinal study, Hijne et al. [40] found only small or negligible associations between changes in content-independent RNT and changes in depression and anxiety over a period of three years. However, symptom severity rather than incidence of full-blown mental disorders was examined, which, again, leaves a lack of knowledge regarding the clinical relevance of these findings. In a longitudinal study testing content-independent RNT in diagnosed mental disorders, Spinhoven et al. [32] demonstrated its predictive value for persistence and relapse of depressive and anxiety disorders. However, these do not allow drawing conclusions regarding the role of *content-independent* RNT to be involved in the *onset* of psychopathology. Finally, few RNT studies explicitly applied a pre-stressor conceptualization of RNT according to the vulnerability-stress perspective. Experimental studies using a laboratory stressor and testing associations with changes in affect and mood strengthen the role of RNT and stress in changes of symptomatology [22, 41]. The lack of research testing associations with stress may mainly be due to the fact that disorder-specific expressions of RNT, particularly rumination and worry, have originally been conceptualized and measured as post-stressor coping strategies following preexisting symptoms, stressful or traumatic events [7, 10]. Furthermore, it is difficult to identify specific stressors for the onset of mental disorder such as anxiety, depression or substance use disorders. However, testing the temporal interplay between *pre-stressor content-independent* RNT, *stress* and the *development of psychopathology* may be important to understand pathways of risk. In the long run, this knowledge might be useful for the development of preventive interventions.

Accordingly, the *second aim* of the present study was to test whether content-independent RNT, assessed prior to a stressor, predicted post-stressor incident psychopathology (i.e., any mental disorder). As an example for stress exposure, we investigated German soldiers before and after military deployment in Afghanistan.

## Methods

Data are part of the longitudinal component of the study “Prevalence, Incidence and Determinants of PTSD and Other Mental Disorders” (PID-PTSD^+3^; [42]) investigating course and risk factors of mental disorders and health problems in soldiers associated with military deployment in Afghanistan. The study protocol has been performed in accordance with the Declaration of Helsinki.

### Design and procedure

**Fig. 1:**
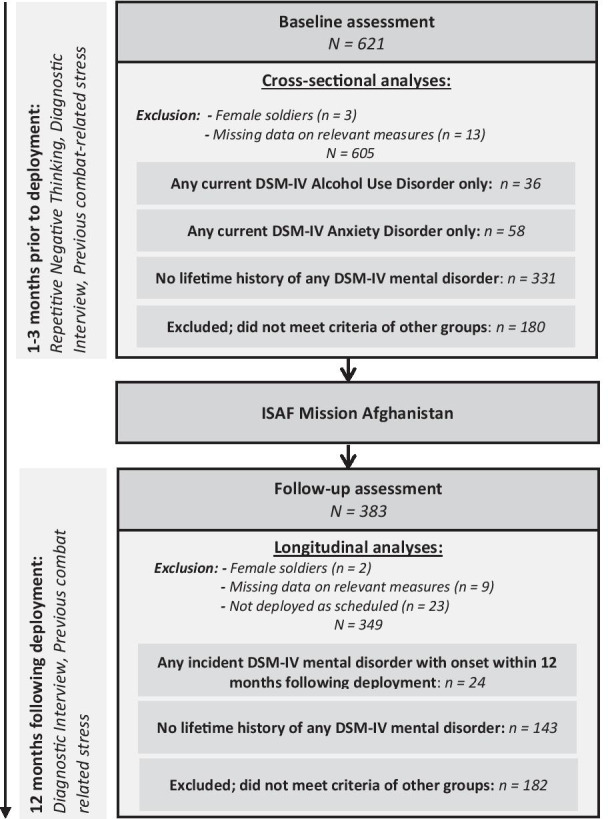
Design and sampling procedure of cross-sectional and longitudinal analyses

Soldiers were examined individually one to three months prior to (baseline) and at least 12 months after deployment (follow-up). For illustration of study design, see Fig. 1. Clinically trained assessors of the Technische Universität Dresden carried out assessments at soldier’s military home base or at private homes. Military personnel and supervisors were blind to study participation. Data collection and processing was conducted in pseudonymized form. Prior to assessments, soldiers were informed about study purpose, assessment procedure and processing of data according to the human study participant’s research ethics approval (University’s Ethics Board, EK 72,022,010). Subsequently, participants were asked for informed consent. Only soldiers who obtained informed consent voluntarily were included in the study. Assessments comprised a standardized diagnostic interview with supplementary questionnaires at the beginning. Then, hair strands were taken and several experimental tasks were conducted for other study purposes (see [42] for details).

### Sample

At baseline 621 soldiers participated to study assessments. Female soldiers (*n* = 3) were excluded from further analyses, because we expected gender related differences in our research questions based on previous research [43] and the small number of females does not allow for adequate subgroup analysis. Finally, we excluded cases with missing data of more than 80% of all items in relevant measures (*n* = 13), which resulted in a total baseline sample of 605 soldiers.

In sum, 383 soldiers participated at the follow-up assessment following deployment. The other participants were either not available anymore (e.g., changes of location or leaving the army) or did not respond. Trautmann et al. [44] tested selective dropouts associated with a history of mental disorders and previous experience of mission combat events at baseline and found no differences between participants and dropouts. Female soldiers (*n* = 2), those with missing values on relevant measures (*n* = 9) and those who were finally not deployed to Afghanistan as scheduled (*n* = 23) were excluded from analyses. The total follow-up sample comprised 349 soldiers. For demographic and clinical characteristics of the total samples at baseline and follow-up, see Tables 1 and 2. Mean duration of deployment in Afghanistan in the total follow-up sample was 5.33 months (*SD* = 1.46).

**Table 1:** Demographic and clinical characteristics of soldiers at baseline

	Total baseline sample	Excluded group	Alcohol use disorders	Anxiety disorders	Healthy group
	(*n* = 605)	(*n* = 180)	(*n* = 36)	(*n* = 58)	(*n* = 331)
Age, mean (*SD*)	26.71 (5.92)	26.59 (5.9)	24.56 (3.68)	26.91 (6.42)	26.97 (6.01)
Education, *n* (%)					
Low	112 (18.54)	44 (24.44)	6 (16.67)	12 (20.69)	50 (15.11)
Middle	400 (66.12)	108 (60)	23 (63.89)	36 (62.07)	233 (70.39)
High	93 (15.37)	28 (15.56)	7 (19.44)	10 (17.24)	48 (14.50)
No. of previous combat experiences, mean (*SD*)	2.38 (4.83)	2.78 (5.33)	1.36 (3.56)	2.91 (5.93)	2.19 (4.42)
PTQ total at baseline, mean (*SD*)	15.27 (9.74)	17.95 (11.78)	16.28 (7.99)	18.64 (9.32)	13.11 (8.11)
PTQ core features, mean *(SD)*	10.30 (6.45)	11.93 (7.59)	11.36 (5.74)	12.33 (6.15)	8.92 (5.53)
PTQ unproductiveness, mean *(SD)*	2.57 (2.20)	3.16 (2.65)*	3.06 (2.07)	3.28 (2.08)	2.07 (1.82)
PTQ mental capacity, mean *(SD)*	2.40 (2.05)	2.84 (2.38)*	1.86 (1.44)	3.03 (2.28)	2.11 (1.80)

PTQ = Perseverative Thinking Questionnaire. SD = Standard deviation. *One missing value. Excluded group (*n* = 180) comprises participants that did not meet criteria of any other group

**Table 2:** Demographic and clinical characteristics of soldiers at follow-up

	Total follow-up sample	Excluded group	Incident disorders group	Healthy group
	(*n* = 349)	(*n* = 182)	(*n* = 24)	(*n* = 143)
Age, mean (*SD*)	28.76 (6.29)	28.35 (5.74)	27.88 (5.98)	29.44 (6.88)
Education, *n* (%)				
Low	66 (18.91)	43 (23.63)	4 (16.67)	19 (13.29)
Middle	224 (64.18)	109 (59.89)	17 (70.83)	98 (68.53)
High	59 (16.91)	30 (16.48)	3 (12.50)	26 (18.18)
No. of previous combat experiences, mean (*SD*)	2.48 (4.84)	2.28 (4.74)	2.92 (5.52)	2.66 (4.88)
No. of intermediate combat experiences, mean (*SD*)	5.31 (5.39)	5.30 (5.60)	8.79 (6.27)	4.74 (4.76)
PTQ total at baseline, mean (*SD*)	15.66 (9.68)	15.67 (9.48)	23.58 (10.75)	14.31 (9.16)
PTQ core features, mean *(SD)*	10.51 (6.40)	10.57 (6.15)	15.59 (7.98)	9.57 (6.04)
PTQ unproductiveness, mean *(SD)*	2.63 (2.23)	2.66 (2.26)	4.46 (2.45)	2.28 (2.00)
PTQ mental capacity, mean *(SD)*	2.52 (2.08)	2.46 (2.02)	3.54 (2.17)	2.44 (2.11)
PTQ total at follow-up, mean (*SD*)	14.86 (10.83)	15.54 (10.53)	25.04 (13.90)	12.26 (9.45)
PTQ core features, mean *(SD)*	10.07 (7.13)	10.40 (6.82)	16.96 (9.05)	8.46 (6.39)
PTQ unproductiveness, mean *(SD)*	2.48 (2.24)	2.67 (2.30)	4.13 (2.88)	1.96 (1.84)
PTQ mental capacity, mean *(SD)*	2.31 (2.18)	2.47 (2.17)	3.96 (2.73)	1.84 (1.91)

PTQ = Perseverative Thinking Questionnaire. SD = Standard deviation. Excluded group (*n* = 182) comprised soldiers who met criteria for one or more lifetime and/or 12-months mental disorders that did not occur first in lifetime within 12 months following deployment

#### Diagnostic groups at baseline

To examine the descriptively transdiagnostic nature of RNT we selected cases fulfilling diagnostic criteria for any current (past 12 month) anxiety disorder including posttraumatic stress disorder (representing a typical expression of the internalizing symptom spectrum) or alcohol use disorders (representing a typical expression of the externalizing symptom spectrum) [35] according to DSM-IV mental disorder [45]. Examining associations of RNT with core characteristics of diagnostic groups, no cases with current or past comorbid disorders of any other than the current diagnostic group were allowed for the two single-disorder-groups, i.e., soldiers of the anxiety disorder group fulfilled diagnostic criteria for any current anxiety disorder, but never fulfilled criteria for any disorder of any other diagnostic group and so on. This resulted in a sample of *n* = 58 participants with anxiety disorders and *n* = 36 participants with current alcohol use disorders. Due to low prevalence rates in our strictly defined single-disorder-groups, cases with any current affective disorder only (*n* = 8) had to be excluded from further analyses. Cases with somatoform disorders only (*n* = 0) and psychotic disorders only (*n* = 0) did not emerge in our sample. A group of soldiers who reported no lifetime history of any mental disorders was included as comparison (Healthy Group, *n* = 331). Participants that did not meet criteria of any group (e.g., lifetime diagnosis but no 12-months diagnosis) were excluded (*n* = 180). For demographic and clinical characteristics of diagnostic groups, see Table 1. Additionally, exploring RNT levels in a comorbid disorder-group, we selected cases who fulfilled diagnostic criteria for two or more current (past 12 month) mental disorders from different diagnostic groups with any combination of lifetime diagnosis allowed. This resulted in *n* = 19 cases with two current mental disorders of the following combination: any anxiety disorder and any affective disorder (*n* = 12); any anxiety disorder and any alcohol use disorder (*n* = 5); any anxiety disorder and any somatoform disorder (*n* = 1), any somatoform disorder and any affective disorder (*n* = 1). Mean PTQ Scores (*SD*) at baseline for the comorbid group were as follows: PTQ total = 28.37 (*11.83*), PTQ core features = 18.00 (*6.91*), PTQ unproductiveness = 5.26 (*2.75*), PTQ use of mental capacity = 5.11 (*2.96*).

#### Incident mental disorders group at follow-up

To examine the predictive value of RNT for the development of psychopathology following deployment, incident cases of mental disorders were identified. Within the total follow-up sample (*n* = 349) we defined incident cases as those fulfilling diagnostic criteria for any mental disorder at follow-up with first onset occurring within 12 months following deployment. This definition allows for identification of incident cases with onset after stress exposure, i.e. deployment to Afghanistan. Twenty-four soldiers fulfilled criteria for any of the following incident mental disorder: Major depressive disorder (*n* = 1), bipolar II disorder (*n* = 4), panic disorder (*n* = 3), agoraphobia (*n* = 8), alcohol abuse (*n* = 3), alcohol dependence (*n* = 1). Three soldiers fulfilled criteria for two incident mental disorders: Pain disorder and generalized anxiety disorder (*n* = 1), agoraphobia and bipolar II disorder (*n* = 1) and PTSD and agoraphobia (*n* = 1). One soldier fulfilled criteria for three new-onset disorders: agoraphobia, alcohol abuse and psychotic disorder. We identified *n* = 143 soldiers who reported to have never fulfilled criteria for any mental disorder at follow-up. Accordingly, the final groups consisted of *n* = 24 in the incident mental disorder group and *n* = 143 in the healthy group including soldiers with no lifetime history of any mental disorders. Soldiers who fulfilled diagnostic criteria for one or more lifetime or 12-months mental disorders with a first onset before the 12-months interval after deployment were excluded from further analyses because these individuals are by definition not at risk for the first onset (i.e. incidence) of a disorder (*n* = 182). For demographic and clinical characteristics of groups, see Table 2.

### Measures

#### Diagnostic status

DSM-IV mental disorders were diagnosed using the computer assisted military version of the Munich-Composite International Diagnostic Interview (DIA-X/M-CIDI [46]). DIA-X/M-CIDI is a standardized clinical interview assessing categorical diagnostic status according to DSM-IV-TR [45] diagnostic criteria. 12-months and lifetime diagnostic status were recorded as well as onset of each disorder.

#### Repetitive negative thinking (RNT)

RNT was measured using the Perseverative Thinking Questionnaire (PTQ; [47]) at baseline, i.e. prior to deployment in Afghanistan. The PTQ is a 15-item self-report instrument measuring content and disorder-independent RNT. Soldiers were asked to indicate the frequency with which they typically engage in RNT about negative events (e.g. “Thoughts come to my mind without me wanting them to.”). Responses are indicated on a 5-point scale ranging from 0 (never) to 4 (almost always). Internal consistency of the PTQ in the present study ranges from 0.89 to 0.91 (Cronbach´s Alpha) for the three diagnostic groups of the cross-sectional sample and is 0.93 for both groups of the longitudinal sample. Three subscores were computed: (i) core features of RNT (9 items; e.g., “thoughts intrude into my mind”), (ii) unproductiveness (three items; e.g., “I think about many problems without solving any of them”) and (iii) use of mental capacity (three items; e.g., “my thoughts take up all my attention”).

#### Assessment of combat related stress

Combat-related stress in former and most recent military deployments was assessed at baseline and at follow-up using a modified list of combat experiences [48, 49]. The list asked to indicate the frequency with which 33 combat-related events (e.g. being attacked or ambushed) have been experienced.

### Statistical analyses

We conducted all analyses with Stata 15.1 [50]. Statistical significance was evaluated two sided at the 5% level. PTQ scores were z-standardized to simplify interpretation.

#### Cross-sectional associations of RNT across diagnostic groups

We used ordinary linear regression models with dummy-coded diagnostic groups as independent variable and PTQ total/subscale scores as dependent variable to test for differences in RNT levels between diagnostic groups (anxiety disorders; alcohol use disorders; healthy group). Additionally, we used a robust estimation of standard errors using the Huber-White-approach [51] to produce confidence intervals that are robust against heteroscedasticity and potential deviations from normal distribution of residuals. To verify that our data meet assumptions of linear regression, we plotted histograms of RNT scores as well as residuals separately for each group to check for normal distribution and expected values of the error terms. Next, we checked for outliers and extreme values by visual inspection of boxplots of dfbeta. Finally, evidence exists showing associations between age and educational level with RNT levels [52, 53], which is why we additionally included these variables. As it seems theoretically conceivable that the amount of previous deployment related stress may be associated with both RNT levels and psychopathology, number of combat related experiences made in former deployments was additionally added as a covariate in separate models. The small sample sizes in the groups did not allow adjustment for all potential confounders in a joint model. Due to small case numbers for the comorbid group, analyses comparing RNT levels of the comorbid group (*n* = 19) with anxiety disorders, alcohol use disorders and healthy participants, were conducted on an exploratory basis. Here, we ran linear regression analyses with dummy-coded diagnostic groups as independent variable and PTQ total/subscale scores as dependent variable.

#### Predictive value of RNT for incident mental disorders

Using logistic regressions, we examined the predictive value of RNT levels (PTQ total score and subscores) prior to deployment for incident mental disorders following deployment. For ease of interpretation, we used the usual approximation of the risk ratio by the odds ratio and confidence intervals, which is possible if incidence proportions are small [54, 55]. Additionally, we inspected the logit-linearity assumption of the logistic regression model by visual inspection of the locally weighted polynomial regression. To check for extraordinary influential observations we looked at leverage [56] and standardized residuals. For proper model specification, again we added age and educational level known to be associated with RNT levels [52, 53] as well as with incident mental disorders [57, 58]. To allow for conclusions about the predictive role of RNT for incident mental disorders above and beyond stress related predictors, we added number of combat related experiences made in former and the most recent deployments into our analyses. Due to small sample size, only one covariate was included into analyses at a time.

## Results

### Cross-sectional associations of RNT across diagnostic groups

Visual regression diagnostics revealed no substantial violations of the normal distribution, only the width of the distributions for each group was slightly increased (i.e., kurtosis below zero), which may have led to slightly too small confidence intervals. However it is known that the influence of non-zero kurtosis on regression inference is very minor [59] Anomalies regarding the plotted residuals in each group were not observed. Boxplots of dfbeta showed no influential outliers or extreme values.

The ordinary linear regression approach and the robust estimation of standard errors revealed similar results. In one group comparison, confidence intervals were even slightly smaller when using robust standard error estimation. Therefore, only the results of the ordinary regression are reported.

**Fig. 2:**
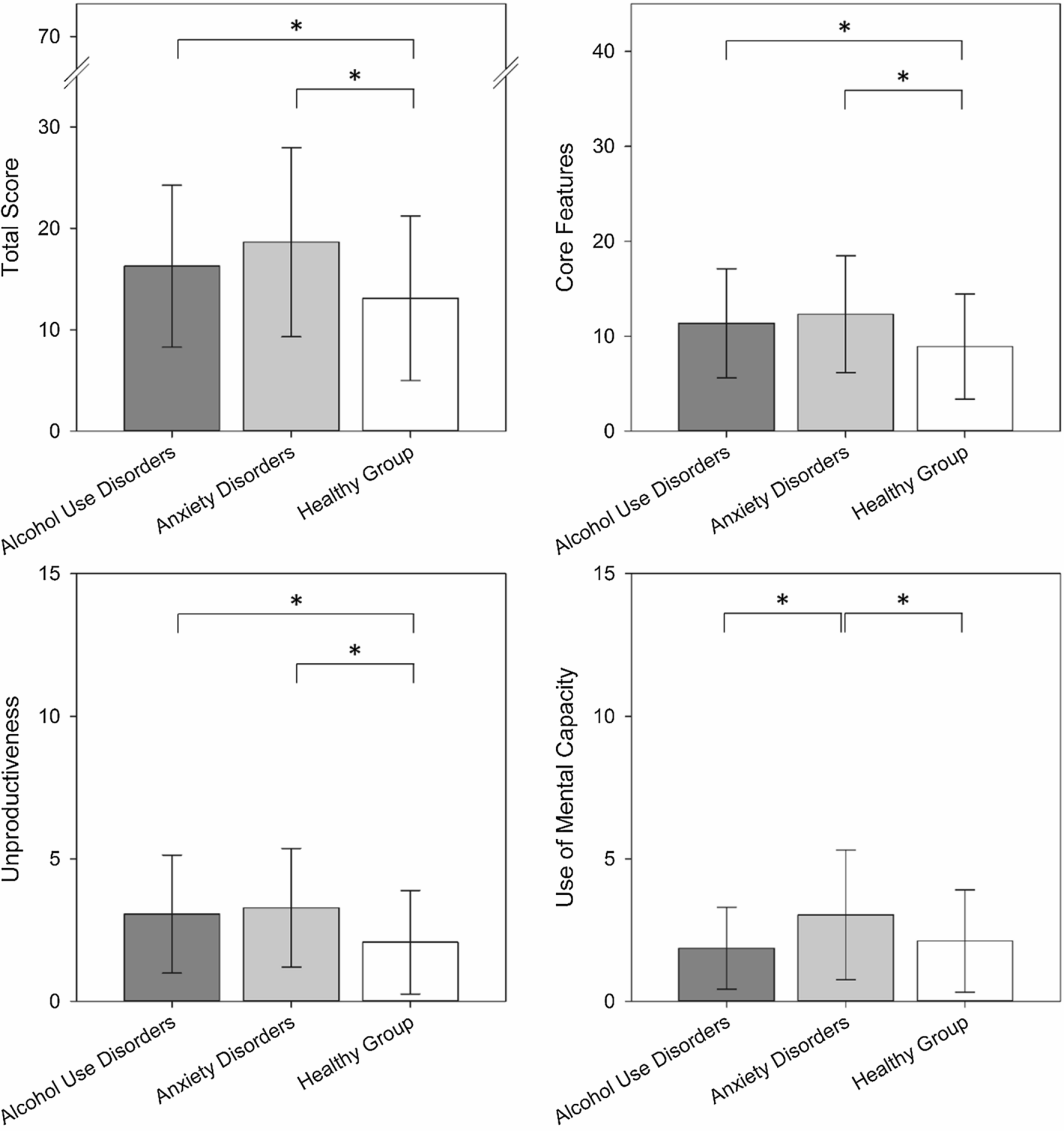
Mean PTQ scores and standard deviations across diagnostic groups and healthy group. Point estimates of group comparisons: PTQ total score: Alcohol Use Disorder-Group versus Healthy Group: *B* = 3.17, 95% CI [0.31–6.02]; Anxiety Disorder-Group versus Healthy Group: *B* = 5.53, 95% CI [3.21–7.84]; PTQ core features: Alcohol Use Disorder-Group versus Healthy Group: *B* = 2.44, 95% CI [0.49–4.38]; Anxiety Disorder-Group versus Healthy Group: *B* = 3.40, 95% CI [1.83–4.98]; PTQ unproductiveness: Alcohol Use Disorder-Group versus Healthy Group: *B* = 0.98, 95% CI [0.33–1.63]; Anxiety Disorder-Group versus Healthy Group: *B* = 1.20, 95% CI [0.68–1.73]; PTQ use of mental capacity: Anxiety Disorder-Group versus Alcohol Use Disorder-Group: *B* = 1.17, 95% CI [0.40–1.94]; Anxiety Disorder-Group versus Healthy Group: *B* = 0.92, 95% CI [0.41–1.44]. * *p* < .05

Regarding the PTQ total score, analyses revealed group differences between each of the diagnostic groups and the healthy group, respectively (see Table 3 and Fig. 2). Specifically, as we expected, pairwise group comparisons showed that RNT was higher in the anxiety disorder-group as well as in the alcohol use disorder-group compared to the healthy group. Diagnosed groups did not differ regarding RNT levels. For PTQ subscales, a similar pattern of results emerged for all subscales except for “use of mental capacity”. Interestingly, only the anxiety disorder-group differed significantly from the healthy group, but not the alcohol use disorder-group (see Table 3). The alcohol use disorder-group however differed from the anxiety disorder-group with those in the anxiety disorder-group reporting higher use of mental capacity.

**Table 3:** Linear regression results of pairwise group comparisons in PTQ scores

		Model I			Model II			Model III			Model IV	
	*β*	95% CI	*p*	*β*	95% CI	*p*	*β*	95% CI	*p*	*β*	95% CI	*p*
*PTQ total score*												
AUD versus Healthy	0.37	[0.04–0.71]	*.030*	0.40	[0.06–0.74]	*.020*	0.37	[0.03–0.71]	*.031*	0.52	[0.09–0.96]	*.019*
AnxD versus Healthy	0.65	[0.38–0.92]	< *.001*	0.65	[0.38–0.92]	< *.001*	0.65	[0.37–0.92]	< *.001*	0.65	[0.31–0.99]	< *.001*
AnxD versus AUD	0.28	[− 0.13–0.68]	*.180*	0.25	[− 0.16–0.66]	*.230*	0.28	[− 0.13–0.68]	*.182*	0.12	[− 0.39–0.64]	*.637*
*PTQ core features *												
AUD versus Healthy	0.42	[0.09–0.76]	*.014*	0.45	[0.11–0.79]	*.009*	0.42	[0.08–0.76]	*.015*	0.44	[0.10–0.76]	*.011*
AnxD versus Healthy	0.59	[0.32–0.86]	< *.001*	0.59	[0.32–0.86]	< *.001*	0.59	[0.31–0.86]	< *.001*	0.59	[0.32–0.86]	< *.001*
AnxD versus AUD	0.17	[− 0.24–0.58]	*.419*	0.14	[− 0.27–0.55]	*.502*	0.17	[− 0.24–0.58]	*.416*	0.15	[− 0.26–0.56]	*.467*
*PTQ unproductiveness*												
AUD versus Healthy	0.51	[0.17–0.84]	*.003*	0.50	[0.16–0.84]	*.004*	0.52	[0.18–0.85]	*.003*	0.52	[0.18–0.85]	*.003*
AnxD versus Healthy	0.62	[0.35–0.89]	< *.001*	0.62	[0.35–0.89]	< *.001*	0.63	[0.36–0.90]	< *.001*	0.63	[0.36–0.90]	< *.001*
AnxD versus AUD	0.11	[− 0.29–0.52]	*.581*	0.12	[− 0.29–0.53]	*.561*	0.11	[− 0.29–0.52]	*.580*	0.11	[− 0.29–0.52]	*.592*
*PTQ mental capacity*												
AUD versus Healthy	− 0.13	[− 0.47–0.21]	*.439*	− 0.08	[− 0.43–0.26]	*.625*	− 0.14	[− 0.48–0.20]	*.419*	− 0.11	[− 0.46–0.22]	*.495*
AnxD versus Healthy	0.49	[0.22–0.77]	< *.001*	0.50	[0.22–0.77]	< *.001*	0.48	[0.20–0.76]	< *.001*	0.47	[0.20–0.75]	< *.001*
AnxD versus AUD	0.63	[0.22–1.04]	*.003*	0.58	[0.17–0.99]	*.006*	0.62	[0.21–1.03]	*.003*	0.59	[0.18–1.00]	*.005*

AUD = Alcohol Use Disorders; AnxD = Anxiety Disorders. Model I: unadjusted, Model II: adjusted for age, Model III: adjusted for education, Model IV: adjusted for number of previous combat related experiences. PTQ score was standardized. *p* < .05 = significant

Exploratory analyses revealed that the comorbid group differed from all other groups (anxiety disorders; alcohol use disorders and healthy participants, respectively) in regard to PTQ total score and all subscores (see Table 4), i.e., the comorbid group had higher RNT levels at all scales compared to the single-disorder-groups and the healthy group.

**Table 4:** Linear regression results of exploratory pairwise group comparisons in PTQ scores for the comorbid disorders-group

	Model I			Model II			Model III			Model IV		
	*β*	95% CI	*p*	*β*	95% CI	*p*	*β*	95% CI	*p*	*β*	95% CI	*p*
*PTQ total score*												
Comorb. versus Healthy	1.67	[1.24–2.10]	< *.001*	1.70	[1.27–2.13]	< *.001*	1.66	[1.23–2.10]	< *.001*	1.68	[1.25–2.11]	< *.001*
Comorb. versus AUD	1.33	[0.81–1.84]	< *.001*	1.33	[0.82–1.84]	< *.001*	1.32	[0.80–1.84]	< *.001*	1.32	[0.80–1.84]	< *.001*
Comorb. versus AnxD	1.07	[0.59–1.55]	< *.001*	1.10	[0.61–1.58]	< *.001*	1.06	[0.58–1.54]	< *.001*	1.08	[0.59–1.56]	< *.001*
*PTQ core features *												
Comorb. versus Healthy	1.50	[1.06–1.94]	< *.001*	1.53	[1.09–1.97]	< *.001*	1.49	[1.05–1.93]	< *.001*	1.51	[1.07–1.94]	< *.001*
Comorb. versus AUD	1.10	[0.57–1.62]	< *.001*	1.10	[0.58–1.62]	< *.001*	1.09	[0.57–1.62]	< *.001*	1.09	[0.57–1.62]	< *.001*
Comorb. versus AnxD	0.94	[0.45–1.43]	< *.001*	0.97	[0.48–1.46]	< *.001*	0.93	[0.44–1.43]	< *.001*	0.94	[0.46–1.43]	< *.001*
*PTQ unproductiveness*												
Comorb. versus Healthy	1.54	[1.12–1.98]	< *.001*	1.54	[1.11–1.98]	< *.001*	1.57	[1.13–2.00]	< *.001*	1.56	[1.12–1.99]	< *.001*
Comorb. versus AUD	1.07	[0.55–1.59]	< *.001*	1.07	[0.55–1.59]	< *.001*	1.08	[0.56–1.60]	< *.001*	1.07	[0.55–1.60]	< *.001*
Comorb. versus AnxD	0.96	[0.48–1.45]	< *.001*	0.96	[0.47–1.45]	< *.001*	0.98	[0.49–1.46]	< *.001*	0.96	[0.48–1.45]	< *.001*
*PTQ mental capacity*												
Comorb. versus Healthy	1.49	[1.05–1.93]	< *.001*	1.54	[1.10–1.97]	< *.001*	1.44	[1.00–1.89]	< *.001*	1.49	[1.05–1.93]	< *.001*
Comorb. versus AUD	1.61	[1.09–2.14]	< *.001*	1.62	[1.09–2.14]	< *.001*	1.57	[1.05–2.10]	< *.001*	1.60	[1.08–2.13]	< *.001*
Comorb. versus AnxD	1.03	[0.54–1.52]	< *.001*	1.07	[0.59–1.57]	< *.001*	1.00	[0.51–1.49]	< *.001*	1.05	[0.56–1.54]	< *.001*

AUD = Alcohol Use Disorders; AnxD = Anxiety Disorders; Comorb. = Comorbid Disorders. Model I: unadjusted, Model II: adjusted for age, Model III: adjusted for education, Model IV: adjusted for number of previous combat related experiences. PTQ score was standardized. *p* < .05 = significant

Finally, results for PTQ total and subscale scores did not substantially differ when adjusting for age (Model II), education (Model III) and number of combat experiences in previous deployments (Model IV), respectively (see Tables 3 and 4).

### Predictive value of RNT for incident mental disorders

**Fig. 3:**
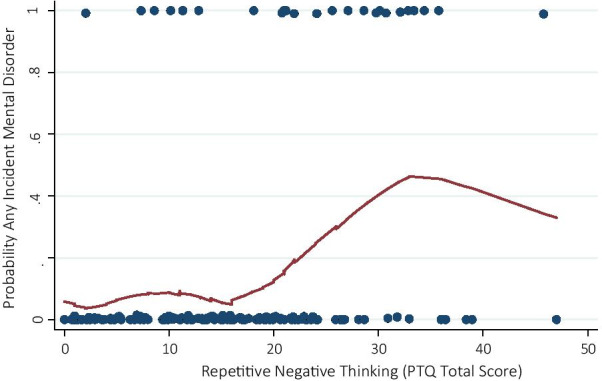
Locally weighted polynomial regression smoother showing predictive association between pre-deployment PTQ total scores and probability for any incident mental disorder following deployment

The dependence of mental disorder incidence probability on PTQ scores prior to deployment was compared to a logistic curve by visual inspection of the locally weighted polynomial regression smoother and revealed satisfying model specification with a caveat. It could be argued that the incidence probability only increases for PTQ scores greater than 15 (see Fig. 3). Hence, we added an additional logistic regression only including subjects with PTQ scores greater than 15 (*n* = 78). In this additional approach, we presume no dependence of incidence probability on PTQ scores below 15. Looking at leverage and standardized residuals we identified no extraordinary influential observations.

Logistic regression analyses revealed that higher RNT levels prior to deployment were associated with higher probability for incidence of any mental disorder during the follow-up period (OR = 2.54; 95% CI [1.58–4.06]; *p* < .001). Accordingly, higher RNT levels by one standard deviation on the PTQ prior to deployment predicted a one and a half to four-fold risk to develop any mental disorder following deployment. Results did not differ when adjusting for age (OR = 2.56; 95% CI [1.59–4.11]; *p* < .001), education (OR = 2.52; 95% CI [1.57–4.04]; *p* < .001), or the amount of total combat experiences made in all previous deployments (OR = 2.52; 95% CI [1.57–4.05]; *p* < .001). Repeating logistic regression analyses on an exploratory basis with only cases above a PTQ score of 15 resulted in an even greater estimated risk ratio (OR = 3.06; 95% CI [1.38–6.77], *p* = .006). This predictive result was mirrored when repeating logistic regression analyses for PTQ subscales (PTQ core features: OR = 2.41; 95% CI [1.53–3.82]; *p* < .001, PTQ unproductiveness: 2.44; 95% CI [1.59–3.76], *p* < .001, PTQ use of mental capacity: OR = 1.64; 95% CI [1.07–2.51]; *p* = .023).

## Discussion

Previous research has shown that disorder-specific RNT variants share many characteristics and are associated with various mental disorders [6]. These findings lead to the theoretical idea that RNT might be a transdiagnostic correlate of psychopathology. Moreover, while theoretically conceivable, there are open questions regarding the involvement of content-independent RNT in the development of psychopathology. In this proof-of-concept study, we tested the transdiagnostic nature of RNT cross-sectionally by comparing RNT in representatives of internalizing (i.e. anxiety disorders) and externalizing (i.e., alcohol use disorders) mental disorders. Additionally, we explored RNT levels in comorbid mental disorders in comparison to healthy participants and participants with a single internalizing (i.e. anxiety disorders) or externalizing (i.e., alcohol use disorders) disorder. Finally, we examined the predictive value of RNT for incident mental disorders longitudinally in a sample of German soldiers pre- and post-deployment to Afghanistan.

In line with our first hypothesis, overall RNT levels were higher in both diagnostic groups compared to the healthy group. Diagnosed groups did not differ from each other regarding RNT levels. The comorbid group however showed significantly higher levels of RNT compared to the healthy participants and to each single-disorder-group. These findings provide further support for content-independent RNT to be transdiagnostically associated with current diagnosed mental disorders even in conceptually distant disorders. Additionally, findings provide evidence that RNT may mirror the severity of psychopathology. The pattern of results for overall RNT was also observed for two of three RNT subcomponents measured by the PTQ, that is “core features of RNT” and “unproductiveness”, but not for the subscale “use of mental capacity.

Further, in order to clarify the role of content-independent and trait-like RNT as a vulnerability factor in accordance with a vulnerability-stress perspective, we examined whether pre-stressor RNT predicts any incident post-stressor mental disorder. In line with our hypothesis, overall RNT levels prior to deployment predicted any incident mental disorder following deployment. Effects were observed incrementally above and beyond number of combat experiences in previous deployments. Similarly, all subcomponents of RNT were predictive for any incident mental disorder. Taken together, results support the theoretical idea that RNT may constitute a vulnerability factor crucially involved in the development of incident mental disorders following exposure to a potentially stressful event. However, due to limited statistical power, we could not test the predictive value of the interaction between RNT and stress on incident mental disorders.

Our findings showing that individuals diagnosed with mental disorders from distant diagnostic spectra both report significantly higher levels of content-independent RNT compared to healthy individuals but do not differ significantly from each other regarding RNT levels provides further support for a transdiagnostic nature of RNT processes, independently of their actual content. This is in line with previous factor-analytic studies showing that disorder-specific RNT expressions share common process [28]. This is also in line with recent findings showing that core processes and characteristics of RNT are higher in multiple clinical groups compared to healthy individuals but do not differ among clinical groups [34]. Cognitive-behavioral theories typically incorporate a disorder-specific cognitive factor thought to be involved in maintenance and aggravation of symptomatology [7–9]. Interestingly, our exploratory finding that the comorbid disorder group showed higher levels of RNT not only in comparison to healthy participants but also compared to both single-disorder-groups may point to a dose–response relation with higher levels of RNT reflecting more severe psychopathology. However, as our findings are based on a small sample size including various mental disorders, replications are highly needed.

Our cross-sectional results of content-independent RNT as a transdiagnostic correlate, together with findings showing that content-independent RNT predicts symptom aggravation [32, 39] are in line with the assumption that content-independent RNT might be transdiagnostically maintaining and aggravating multiple symptomatologies. Interestingly, the pattern of results for cross-sectional group comparisons was not found for the RNT subcomponent “use of mental capacity”. Here, the alcohol use disorder-group reported RNT levels that were not different compared to the healthy group and lower compared to the anxiety disorder-group. Thus, individuals with alcohol use disorders seem not to be caught up by their repetitive thoughts as much as individuals with anxiety disorders, which might be explained by lower abilities of sustained attention in alcohol-dependent individuals [60]. At the same time, they experience repetitive, intrusive and uncontrollable thoughts and their unproductiveness to a similar degree compared to those suffering from anxiety disorders. Further research may investigate underlying mechanisms of these specific associations.

Practically, our results may imply that in treatment seeking individuals, assessment of content-independent overall RNT may help to inform about one central maintaining or aggravating factor across psychopathologies early in the treatment process. The present study contributes to the literature on RNT by first using a measure of content-independent RNT to address comparability issues arising from disorder-specific measures of RNT [47]. Second, content-independent RNT levels were examined simultaneously in two or more diagnosed groups [32, 34]. Third, RNT levels were examined in exclusive diagnostic groups, that is, participants had no current or past comorbidities of any other than the currently fulfilled diagnostic group. Fourth, research on the transdiagnostic nature of psychological constructs is mostly conducted using depression and anxiety outcomes, which indeed share many commonalities [61]. By demonstrating heightened RNT levels in individuals diagnosed with alcohol use disorders and anxiety disorders compared to healthy individuals we add some further evidence for the transdiagnostic nature of content-independent RNT.

Our findings regarding the predictive value of RNT and its subcomponents for incident mental disorders provide support that trait-like and content-independent RNT may indeed be crucially involved in the development of incident mental disorders. Most interestingly, we find evidence that the probability for any incident mental disorder is nearly zero within lowest RNT levels but begins to increase at a PTQ score of 15. The fact that, in our observational longitudinal approach, RNT was assessed explicitly prior to a stressor, i.e. deployment to Afghanistan, suggests that RNT acts a pre-stressor vulnerability factor rather than a reactive process following a stressful event. As we adjusted for previous stressful combat-related events it is unlikely that the predictive association between RNT and incident mental disorders was simply a result of those stressful events. However, we were not able to adjust for all kind of previous and current stressors that might have promoted RNT in the first place. Thus, we cannot fully rule out that pre-deployment RNT might still be an expression of stressful events, i.e. participants may have experienced any other non-deployment related stress before that may influence our findings by some (not trauma-related) “building block effect” [62]. Alternatively, findings might be due to a momentary expression of anticipatory stress facing upcoming deployment [63].

In any case, if the conceptualization of a transdiagnostic and trait-like RNT holds true, it may be of particular interest to identify factors that lead to heightened levels of trait RNT in the first place. Mechanisms currently discussed are childhood adversities [64] or emotion regulation deficits [17]. Taken together, our predictive findings strengthen the role of RNT as a trait-like cognitive processing style representing a distal cognitive risk marker that may inform about future mental health risks. Practically, our findings strengthen the role of content-independent RNT as a promising candidate for prevention and intervention programs. This may particularly apply for populations at risk to experience psychologically distressing events. Finally, future studies should examine the predictive value of content-independent RNT across multiple groups of incident mental disorders in order to provide evidence that content-independent RNT is transdiagnostically involved in the development of psychopathology [65]. Identifying such mechanistically transdiagnostic constructs is of particular interest as these may qualify as potent targets for transdiagnostic prevention and treatment programs [66].

Our study has several limitations. We conducted analyses in a very specific sample of German soldiers prior to and following deployment in Afghanistan. Accordingly, generalizability to other populations and stressors may be limited. Further, our cross-sectional analyses were restricted to two diagnostic groups, i.e. anxiety and alcohol use disorders. Thus, it cannot be ruled out that RNT predicts its risk for psychopathology in dependence of these special conditions [67]. Cross-sectional group comparisons for comorbid cases were only based on 19 individuals and should therefore be interpreted with caution. Next, our sample included only male individuals. However, known gender differences in disorder-specific forms of RNT are usually characterized by lower levels of RNT in male participants compared to females [43]. Consequently, we expect that if there is a gender bias in our findings, we rather underestimate than overestimate associations between RNT and risk for incident mental disorders. Further, our predictive analyses comprised a small number of individuals that reported incident mental disorders following deployment to Afghanistan. Therefore, we could not conduct separate predictive analyses that allow distinguishing between different incident diagnostic groups.

## Conclusion

Our results strongly support the theoretical assumption of content-independent RNT as not only a transdiagnostic correlate, but a vulnerability factor for incident psychopathology. To the best of our knowledge, this is the first study to demonstrate the predictive value of content-independent RNT assessed prior to distressing events for post-stressor and first in lifetime mental disorders. Our findings have important practical implications as RNT may qualify as a potent distal indicator for future mental health risks and may thus be a promising candidate to be targeted in prevention and treatment programs.

## Data Availability

The datasets generated and analyzed during the current study are not publicly available due to privacy restrictions (i.e., pseudonymized data collection and no informed consent about public availability of the raw data from the participants during data collection in 2011–2012) but are available from the corresponding author (Judith Schäfer) on reasonable request.
